# Fidaxomicin Use for Clostridium Difficile Infection Probably Decreases the Effect of Coumadin® in Elderly

**DOI:** 10.7759/cureus.11915

**Published:** 2020-12-05

**Authors:** Samyak Dhruv, Abhishek Polavarapu, Vivek Gumaste

**Affiliations:** 1 Internal Medicine, Staten Island University Hospital, Staten Island, USA; 2 Gastroenterology, Staten Island University Hospital, Staten Island, USA

**Keywords:** fidaxomicin, coumadin, clostridium difficile infection

## Abstract

The incidence of *Clostridium difficile* infection (CDI) has been decreasing in the last decade, though the incidence of community-acquired CDI has remained stable. In an elderly patient on Coumadin®, we report an unexpected decrease in international normalized ratio (INR) during the treatment of second recurrence of CDI treated with fidaxomicin. According to the available information, fidaxomicin does not interfere with warfarin. However, in this case, warfarin effects diminished, and only with increased dosage therapeutic INR was achieved.

## Introduction

Fidaxomicin has been approved for the use of the initial episode of non-severe and severe *Clostridium difficile* (C.diff) infection (CDI) [1], though in clinical practice vancomycin is used for initial episodes and fidaxomicin is used for the recurrent episodes of C.diff more often. Recurrent episodes of C.diff can also be treated by the pulsed taper regimen of vancomycin. We report an unexpected decrease in international normalized ratio (INR) in a patient on Coumadin® treated with fidaxomicin for recurrent C.diff.

This case report was also presented as a poster at ACG 2020 (P0513 (S1635): Fidaxomicin Use for Clostridium Difficile Infection Decreases the Effect of Coumadin in Elderly.

## Case presentation

**Figure 1:**
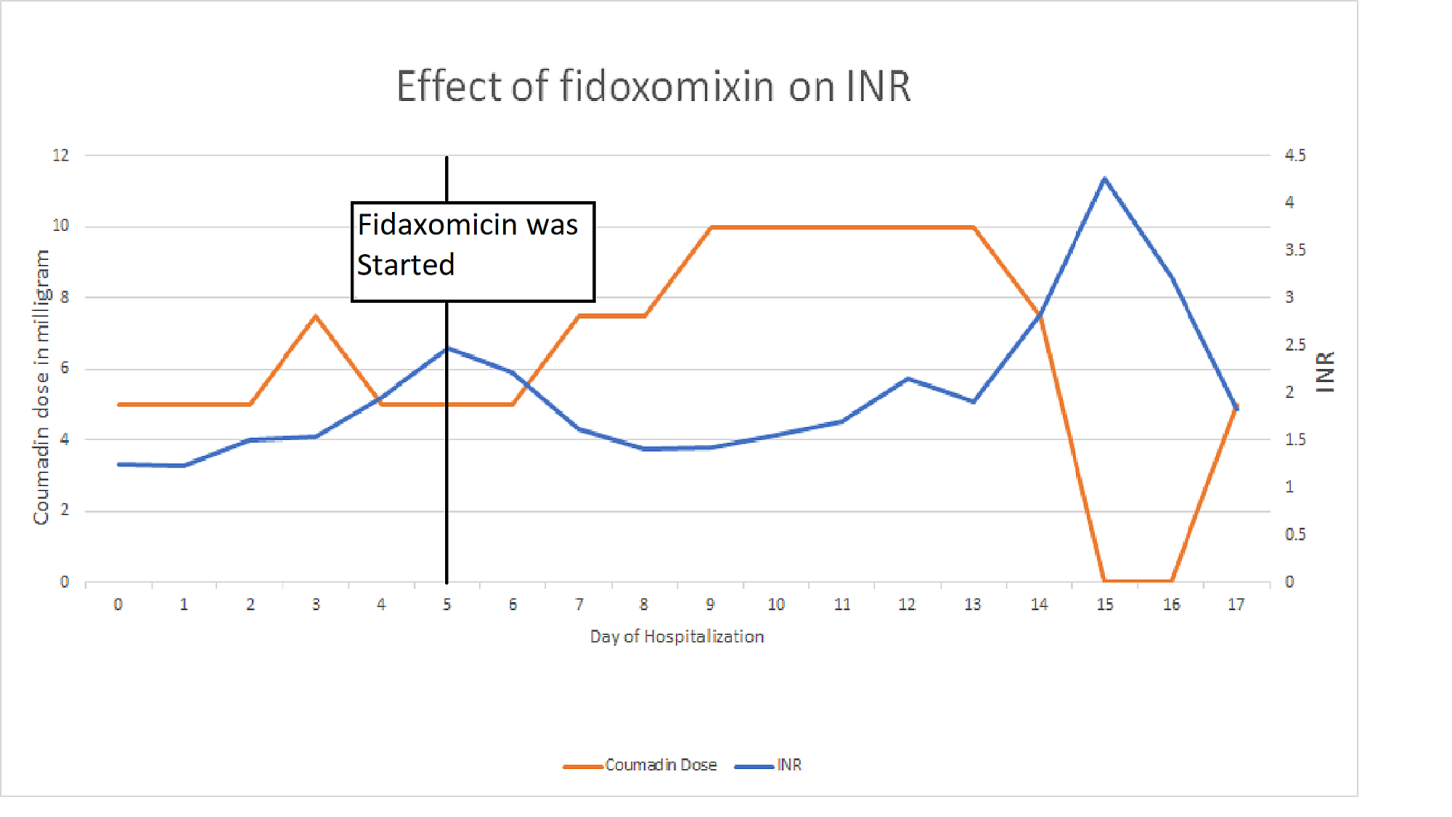
Graph showing the effect of fidaxomicin on INR in our patient. INR, international normalized ratio

A 66-year-old female with past medical history of end-stage renal disease on dialysis and with hypertension, diabetes, dyslipidemia, CDI a year ago, and right internal jugular vein thrombus on Coumadin presented with diffuse abdominal pain, nausea, and diarrhea occurring approximately five to seven times per day (liquid in consistency). Her previous episode of C.diff was treated with vancomycin a year ago. On admission, other laboratory studies were normal except creatinine of 7.2 and blood urea nitrogen of 34. The patient received dialysis on the day of the admission. All the vitals were normal on admission. Stool specimen was sent, and C.diff PCR came positive. White blood cell count stayed around 5,000/microliter during the admission; therefore, it was classified as a nonsevere episode of C.diff. The patient was started on the fidaxomicin therapy as this was her first recurrence, and the initial episode was treated with vancomycin. From the next day of starting fidaxomicin, INR started declining (Figure 1); it became subtherapeutic requiring more Coumadin dose reaching to 10 mg per day from baseline of 3-5 mg per day. Apart from fidaxomicin and Coumadin, the patient was on metoprolol, nifedipine, insulin, and atorvastatin during the hospital stay, none of which was proven to cause the subtherapeutic INR. The patient remained on the same low salt, low fat diet during the hospital stay, ruling out any diet-related alteration in INR. The patient remained in the hospital for almost two weeks for severe diarrhea, subtherapeutic INR, and eventually supratherapeutic INR. Naranjo et al. adverse drug reaction probability scale [2] indicated a probable association (score of 7) between fidaxomicin use and patient’s decreased INR.

## Discussion

From 2003 to 2006, CDI was observed to be more frequent, refractory to standard therapy, and more likely to be relapsed than previously described [3]. It was due to a new strain of C.diff named NAP1/B1/027, which produces binary toxin, which is absent in the other strains of routine C.diff [4]. This strain of C.diff is highly virulent, and patients aged 60-90 years infected with this NAP1 strain are twice as likely to die compare to if infected with non-NAP1 strain [5]. However, the incidence of the CDI has decreased from 2011 to 2017 due to decrease in the rates of hospital-acquired infections, though the rate of community-acquired infections has remained the same [6]. Unusually, severe CDI has also been reported in the peripartum women and in the other healthy individuals without any history of the recent antibiotics use or recent hospitalization [7]. Guidelines recommend fidaxomicin as a treatment option for the severe or non-severe initial episode of CDI, first recurrence (if vancomycin given for the initial episode), and second or subsequent recurrence [1]. Fidaxomicin has been significantly more effective than vancomycin if other concurrent antibiotics are used for other indications and preventing recurrences regardless of other antibiotics use [8]. Also, fidaxomicin oral formulation has been approved by the FDA in January 2020 for CDI in patients older than six months. This formulation would be available in the market from October 2020. Due to these reasons, fidaxomicin use is anticipated to increase in the upcoming future. Therefore, it is very important for gastroenterologists and infectious disease doctors to be aware of the side effects of it. Most commonly observed side effects with the use of the fidaxomicin are nausea, skin rash, and elevated liver enzymes.

Limitations of this case report include the absence of a fidaxomicin rechallenge. Extensive literature search was performed on multiple databases, and to the best of our knowledge there is only one article on this interaction before this. As gastroenterologists increase the use of this drug to treat CDI, further studies need to be conducted to confirm this interaction and to prevent catastrophic complications in the elderly with subtherapeutic INR.

## Conclusions

Because of the possibility of decreased INR and Coumadin failure with the use of fidaxomicin, we recommend a careful use of fidaxomicin in C.diff when a patient is on Coumadin and at high risk for clots. INR should be monitored daily to ensure that adequate Coumadin doses are given. Alternate regimen such as the prolonged pulse taper of vancomycin should be considered for the C.diff recurrences when a patient is on Coumadin therapy.
